# Community-Based Outbreaks in Vulnerable Populations of Invasive Infections Caused by *Streptococcus pneumoniae* Serotypes 5 and 8 in Calgary, Canada

**DOI:** 10.1371/journal.pone.0028547

**Published:** 2011-12-27

**Authors:** Otto G. Vanderkooi, Deirdre L. Church, Judy MacDonald, Franziska Zucol, James D. Kellner

**Affiliations:** 1 University of Calgary, Calgary, Canada; 2 Alberta Children's Hospital, Alberta Health Services, Calgary, Canada; 3 Calgary Laboratory Services, Calgary, Canada; 4 Public Health Portfolio, Alberta Health Services – Calgary Zone, Calgary, Canada; Health Protection Agency, United Kingdom

## Abstract

**Background:**

Outbreaks of invasive pneumococcal disease (IPD) typically occur within institutions. Beginning in 2005, we detected an increase in serotype (ST) 5 and ST8 IPD cases, predominantly in homeless persons living in an open community.

**Methodology/Principal Findings:**

CASPER (Calgary Area S. pneumoniae Epidemiology Research) surveillance study of all IPD (sterile site isolates) in our region (pop ∼1,100,000). Interviews and chart reviews of all cases and all isolates phenotypically analyzed and selected isolated tested by multi-locus sequence typing (MLST).

**Conclusions/Significance:**

During 2005–2007, 162 cases of ST5 IPD and 45 cases of ST8 IPD were identified. The isolates demonstrated phenotypic and genotypic clonality. The ST5 isolates were sequence type (ST) 289 and demonstrated intermediate susceptibility to TMP-SMX. The ST8 isolates were predominantly ST1268, with a susceptible antimicrobial susceptibility profile. Individuals with ST5 IPD were more likely to be middle aged (OR 2.6), homeless (OR 4.4), using illicit drugs(OR 4.8), and asthmatic(OR 2.6). Those with ST8 were more likely to be male (OR 4.4), homeless (OR 2.6), aboriginal (OR7.3), and a current smoker (OR 2.5). Overlapping outbreaks of ST5 and ST8 IPD occurred in an open community in Calgary, Canada and homelessness was a predominant risk factor. Homelessness represents a unique community in which pneumococcal outbreaks can occur.

## Introduction

*Streptococcus pneumoniae* (pneumococcus) is a leading cause of morbidity and mortality worldwide. Bacteremia, meningitis and pneumonia are the most common manifestations of invasive pneumococcal disease (IPD). Otitis media, sinusitis and non-bacteremic pneumonia are the most common noninvasive forms.

Outbreaks due to pneumococcus have been most frequently described in “closed” institutional settings,with the presence of crowding (hospital wards, nursing homes, day care centers) [1], [2], [3], [4], [5], [6], [7]. Some pneumococcal serotypes are more frequently associated with outbreaks, including serotypes 1, 2, 4, 5, 9V, 12F, 14 and 23F [8].

We describe two large outbreaks of IPD in Calgary, Alberta, caused by serotypes 5 and 8 (ST5, ST8. These outbreaks took place in the community, but preferentially affected specific populations. The early features of these outbreaks were previously reported [9], [10]. The ST5 outbreak spread across western Canada and some features have been reported elsewhere [11], [12], .

## Methods

### Outbreak investigation

Active, population-based surveillance of IPD has been conducted in the city of Calgary and surrounding area since January 1, 1998 by the Calgary Area *Streptococcus pneumoniae* Epidemiology Research (CASPER) team [14]. The area is an integrated, publicly funded health region (Calgary Health Region, CHR) that includes the hospitals and outpatient facilities within the city of Calgary, as well as medical centres serving rural areas surrounding the city (population 1,111,614 in 2007).

Patients were deemed to be CHR residents on the basis of home postal code listed in the admission and laboratory records. If this information was unavailable, patients with provincial health care numbers were considered to be CHR residents if the culture specimen was submitted to a collection site within the CHR boundaries. This study was approved by the Conjoint Health Research Ethics Board of the University of Calgary and written, informed consent was obtained from persons with IPD were enrolled in the study.

Cases of IPD (infections with positive cultures from normally sterile body fluids) are identified through active laboratory surveillance in Calgary Laboratory services (CLS), a centralized laboratory service that provides clinical microbiology services to all hospitalized and ambulatory patients in the CHR. Although IPD is a notifiable disease in Alberta requiring notification of local public health officials of all cases to permit appropriate public health follow-up and reporting (passive surveillance), the CASPER surveillance project is separate from public health and collects more detailed information on cases and organisms.

For all IPD cases since 2003, a patient interview was conducted as well as a detailed chart review for all cases for 30 days after the diagnosis. Demographic and pre-existing health status information was collected, as were details of the clinical course and outcome.

Increased cases of serotype specific IPD (serotypes 5 and 8) were identified, starting in 2005 for ST8 and 2006 for ST5. These cases occurred only in adults so the analysis in this manuscript includes only persons aged 16 years or more.

The cases of ST5 IPD occurred frequently in the homeless population and a targeted intervention was directed to try to control transmission. A one-week vaccination campaign, with the 23-valent pneumococcal polysaccharide vaccine (PPV23), was conducted by the CHR Public Health program in December 2006. The PPV23 was offered at several homeless shelters and agencies. Approximately 655 doses of vaccine were administered. In the autumn of 2006, before the outbreak, 339 doses of pneumococcal vaccine had been provided to the homeless population, as that group was newly considered eligible for vaccine in the province. The outbreak was determined to be over 5–6 months later when the number of cases returned to baseline.

During, the vaccine campaign, a survey to determine nasopharyngeal colonization with pneumococcus, with a brief questionnaire, was performed at the main homeless shelter in Calgary. Pneumococcal isolates obtained from this survey were also tested for antibiotic susceptibility and serotype.

### Definitions

Aboriginal status was determined by patient interview and chart review.

Sepsis was defined as a systemic response to infection in the context of confirmed evidence of IPD. The systemic response was manifested by two or more of the following: temperature >38°C or <36°C, white blood cell count >12,000/µL, </µL 4000/µL or 10% immature forms (bands), heart rate >90 beats per minute or a respiratory rate >20 breaths per minute or arterial CO_2_ tension <32 mmHg.

Septic shock was defined as sepsis associated with organ dysfunction: hypotension despite adequate fluid resuscitation and requiring use of vasoactive drugs, defined by systolic blood pressure greater than or equal to 90 mmHg; metabolic acidosis; oliguria; hypoxia, not explained by primary respiratory distress; confusion; renal impairment, creatinine >/ = to 177 µmol/L outside the context of pre-existing renal disease; coagulopathy, evidenced by platelets ≤100,000/mm^3^×10^6^/L, and/or evidence of disseminated intravascular coagulation (defined by prolonged clotting times,) low fibrinogen level, and/or the presence of five and degradation products); liver involvement, alanine aminotransferase (ALT), aspartate aminotransferase (AST), total bilirubin greater than or equal to twice the upper limit of normal; or acute respiratory distress syndrome.

### Laboratory methods

Isolates were confirmed to be pneumococcus by standard methodology including Gram stain, colonial morphology on blood agar, bile solubility, susceptibility to Optochin and pneumococcal antibody agglutination (Phadebact Pneumococcus, Boule Diagnostic AN, Sweden). Susceptibility testing was performed by broth microdilution (PML Microbiologicals, bioMérieux Canada, Inc.St. Laurent , QC, Canada) and results were interpreted according to Clinical and Laboratory Standards Institute (CLSI) guidelines [15], [16], [17], [18].

For the NP colonization survey at the homeless drop-in centre, a single NP swab was obtained from each participant, using the WHO recommended procedure [19]. Questionnaires were administered by trained nursing personnel.

Serotypes were determined by the Quellung reaction, with use of commercial antisera obtained from the Statens Seruminstitut (Copenhagen, Denmark) at the National Centre for Streptococcus (Edmonton, Canada). Five isolates of each ST5 and ST8 (chosen from the chronologic list of isolates analyzing the first, last and the beginning of each quartile) were analyzed by multilocus sequence typing (MLST) at CLS. The sequences of the internal fragments of the 7 housekeeping loci used in the pneumococcal MLST scheme were determined for each isolate, as described elsewhere [20].

### Data Analysis

Data were analyzed using SPSS 16.0 [SPSS Inc., Chicago] and SAS 9.2 (SAS Institute Inc., Cary, NC, USA). Categorical data were summarized as proportions and continuous data were summarized as means with standard deviations (SD). Comparisons were made between IPD cases caused by serotypes 5 and 8 and IPD cases caused by other serotypes. Differences between groups were tested by the Chi-Square test and Fisher's exact test for categorical variables and the Student's t-test for continuous variables. Multivariate logistic regression was performed to determine factors associated with either ST5 or ST8 cases over the study period. Only those traits that were possibly significant (P≤0.10) in univariate analysis were included in the multivariate model. Significance in the multivariate model was determined by a p-value of <0.05. Odds rations (OR) and 95% confidence intervals (95% CI) are reported.

## Results

**Figure 1:**
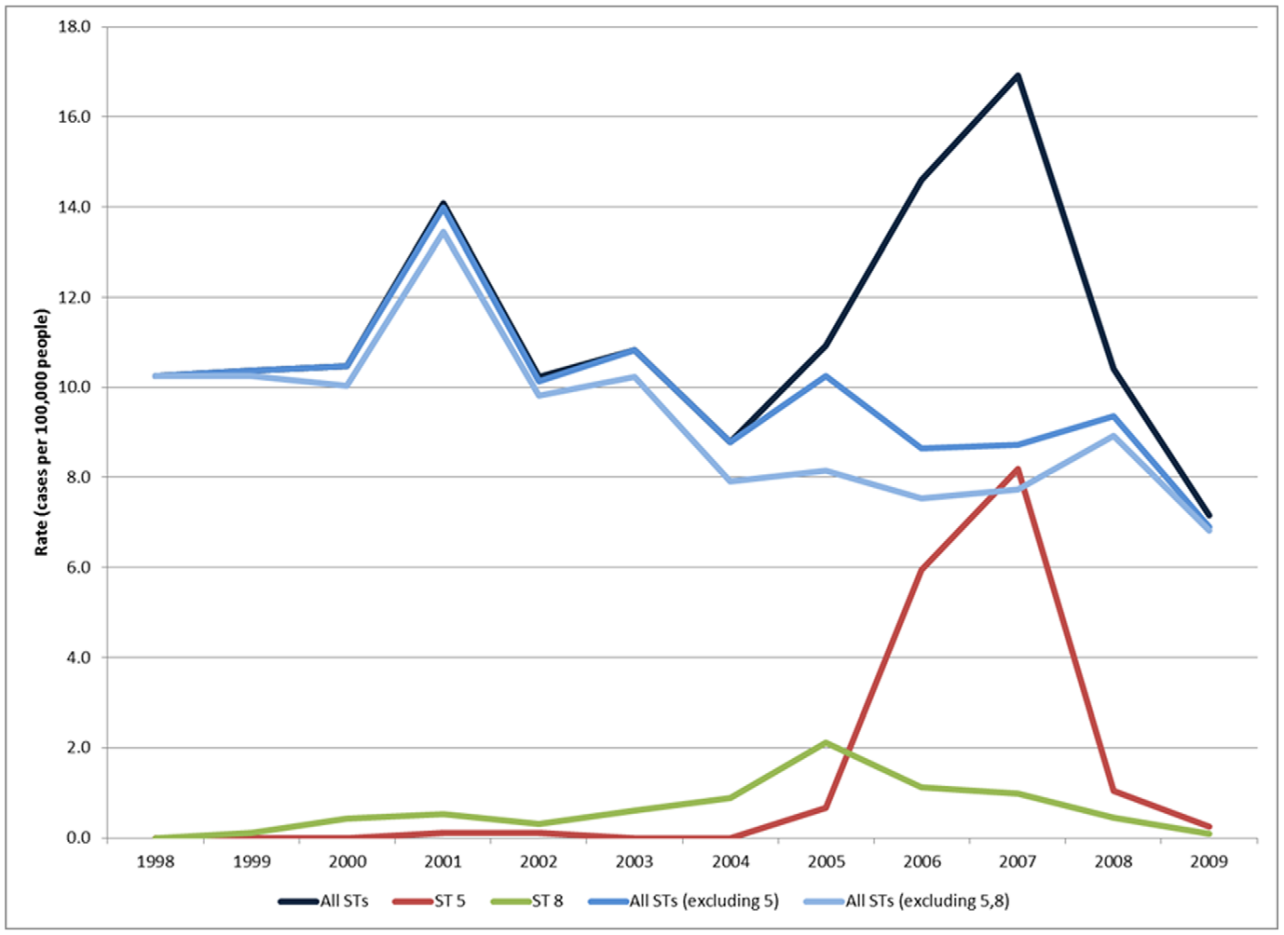
Incidence of Invasive *Streptococcus pneumoniae* Infections, highlighting outbreaks with serotype 5 and 8, in the Calgary Health Region, 1998–2009.

**Figure 2:**
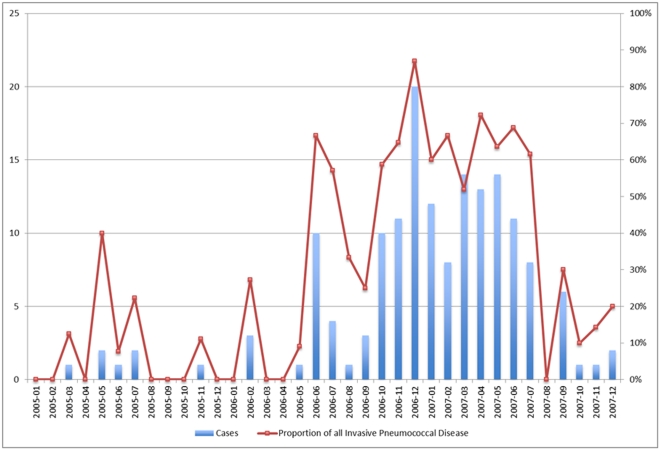
Epidemic Curve of *Streptococcus pneumoniae* Serotype 5 Outbreak in Patients Greater Than 16 Years of Age, Calgary Health Region, 2005–2007.

**Figure 3:**
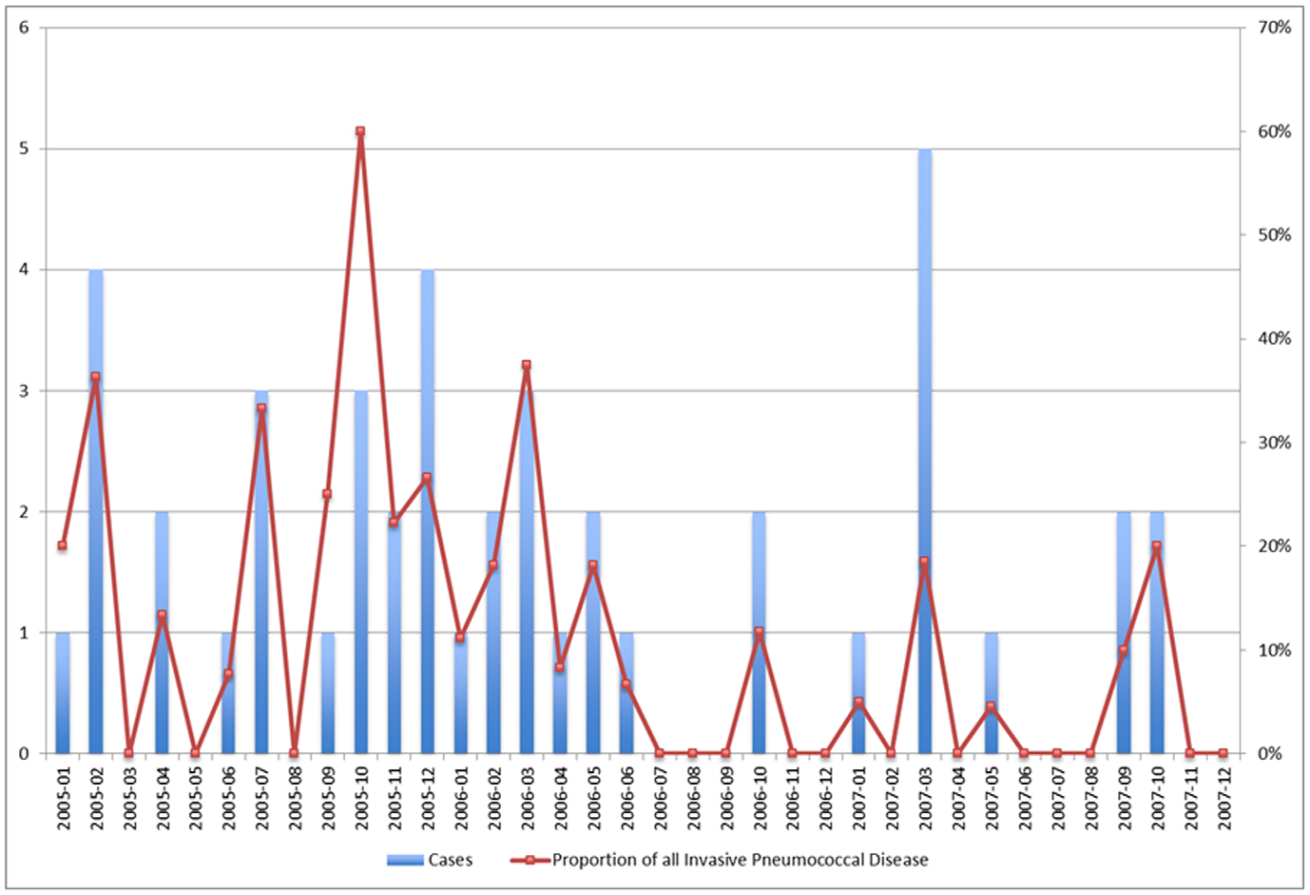
Epidemic Curve of *Streptococcus pneumoniae* ST8 Outbreak in Patients Greater Than 16 Years of Age, Calgary Health Region, 2005–2007.

A total of 1382 laboratory confirmed cases of IPC occurred in the CHR from January 1998 to December 2008. There was an average of 118 cases per year (Table 1). The annual incidence of IPD is shown in Figure 1. Figures 2 and 3 show the number of cases of serotypes 8 and 5 IPD monthly, respectively, for the defined outbreak period from 2005 to 2007. During this period, 162 cases of IPD due to ST5 and 45 cases of ST8 occurred.

**Table 1:** Total Annual Cases of Invasive Pneumococcal Disease (IPD) and ST5 and ST8 IPD Cases from 1998 to 2008, Calgary, AB.

Year	Total IPD Cases (n)	ST5 IPD Cases (n,%)	ST8 IPD Cases (n,%)
**1998**	91	0 (0.0)	0 (0.0)
**1999**	95	0 (0.0)	1 (1.0)
**2000**	98	0 (0.0)	4 (4.1)
**2001**	135	1 (0.7)	5 (3.7)
**2002**	101	1 (1.0)	3 (3.0)
**2003**	109	0 (0.0)	6 (5.5)
**2004**	90	0 (0.0)	9 (10.0)
**2005**	114	7 (6.1)	22 (19.3)
**2006**	157	64 (40.8)	12 (7.6)
**2007**	188	91 (48.4)	11 (5.8)
**2008**	119	12 (10.1)	5 (4.2)
**2009**	85	3 (3.5)	1 (1.2)
**Total**	1382	179 (12.9)	79 (5.7)

Table 2, 3, 4 describes the features of ST5 and ST8 IPD cases from 2005 to 2007, each compared with all other IPD cases from 2000 to 2007, in persons >/ = 16 years of age.. Table 5 describes the multivariate logistic regression analysis of demographic features, risk conditions and outcomes associated with serotypes 5 and 8 cases for all cases where complete was available.

**Table 2:** Demographic features of IPD in patients 16 years of age or older before, during and after serotype 5 & 8 outbreaks, Calgary Health Region 2000–2007, univariate analysis.

	All IPD (less ST5 & ST8) (2000–2007) n (%) , N = 596	Outbreak ST5 cases (2005–2007) n (%), N = 160	P Value (Fisher's) -ST5^1^, OR (95% CI)	Outbreak ST8 cases (2005–2007) n (%), N = 44	P Value (Fisher's) - ST8^2^, OR (95% CI)
**Demographics**					
**Full clinical review**	586 (98%)	156 (98%)		44 (100%)	
**Sex (male)**	319 (54%)	106 (68%)	1.8 (1.2–2.6)	36 (82%)	3.8 (1.7–8.4)
**Age 16–64 years**	380 (65%)	150 (96%)	13.6 (5.9–31.2)	36 (82%)	2.9 (1.3–6.5)
**Homeless**	55 (9%)	82 (53%)	10.7 (7.0–16.3)	19 (43%)	7.3 (3.8–14.2)
**Aboriginal**	36 (6%)	23 (14%)	2.6 (1.5–4.6)	15 (34%)	7.9 (3.9–16.1)

1 Compared to all IPD less ST5 & ST8.

2 Compared to all IPD less ST5 & ST8.

**Table 3:** Clinical features and Outcome of IPD in patients 16 years of age or older before, during and after serotype 5 & 8 outbreaks, Calgary Health Region 2000–2007, univariate analysis.

	All IPD (less ST5 & sT8) (2000–2007) n (%)	Outbreak ST5 cases (2005–2007) n (%)	P Value (Fisher's) – ST5^3^	Outbreak ST8 cases (2005–2007) n (%)	P Value (Fisher's) - ST8^4^
**Inpatient**	535 (91%)	138 (88%)	0.05^5^	35 (80%)	0.01^6^
**Emergency visit only**	40 (7%)	11 (7%)		5 (11%)	
**Treated as outpatient**	5(1%)	6 (4%)		2 (5%)	
**Never seen at hospital**	6 (1%)	1 (1%)		2 (5%)	
**Meningitis**	33 (6%)	1 (1%)	0.05^7^	3(7%)	0.87^8^
**Pneumonia**	476 (81%)	151 (97%)		37 (84%)	
**Bacteremia only**	62 (11%)	3 (2%)		3 (7%)	
**Other IPD** 9	15 (3%)	1 (1%)		1 (2%)	
**Sepsis 1st 72 hrs**	565 (96%)	152 (97%)	ns	42 (95%)	ns^10^
**Severe sepsis**	260 (44%)	75 (48%)	ns	20 (45%)	ns
**Septic shock**	49 (8%)	9 (6%)	ns	2 (5%)	ns
**Outcome**			**P Value**		**P Value**
**Mean hospital days**	13.1 (0–233)	11.8 (0–72)	ns	10.3 (0–138)	ns
**Discharged home**	361 (62%)	102 (65%)	<0.01^11^	23 (52%)	<0.01^12^
**Left against medical advice**	15 (3% )	14 (9%)		5 (11%)	
**Died – IPD** 13	84 (14%)	5 (3%)		3 (7%)	

3 Compared to all IPD less ST5 & ST8.

4 Compared to all IPD less ST5 & ST8.

5 X^2^ test only, for treatment location.

6 X^2^ test only, for treatment location.

7 X^2^ test only, for clinical syndrome.

8 X^2^ test only, for clinical syndrome.

9 Other IPD = sterile site culture other than CSF, pleural or Blood.

10 not significant (P>0.15).

11 X^2^ test only, for disposition.

12 X^2^ test only, for disposition.

13 30 day mortality.

**Table 4:** Risk Factors of IPD in patients 16 years of age or older before, during and after serotype 5 & 8 outbreaks, Calgary Health Region 2000–2007, univariate analysis.

	All IPD (less ST5 & ST8) (2000–2007) n (%), N = 596	Outbreak ST5 cases (2005–2007) n (%), N = 160	OR (95% CI)	Outbreak ST8 cases (2005–2007) n (%), N = 44	OR (95% CI)
**Current Smoker** 14	328 (56%)	129 (83%)	3.8 (2.4–5.9)	40 (91%)	7.9 (2.8–22.3)
**Alcoholism**	124 (21%)	88 (56%)	4.8 (3.3–7.0)	21 (48%)	3.4 (1.8–6.4)
**Illegal Drugs**	60 (10%)	84 (54%)	10.2 ( 6.8–15.5)	14 (32%)	4.1 (2.1–8.1)
**OTC Drugs**	7 (1%)	3 (2%)	1.6 (0.4–6.4, ns)	0	
**HIV/AIDS**	22 (4%)	14 (9%)	2.5 (1.3–5.1)	3 (7%)	1.9 (0.5–6.5)
**Hepatitis A or B**	6 (1%)	6 (4%)	3.9 (1.2–12.2)	1 (2%)	2.3 (0.3–19.1)
**Hepatitis C**	51 (9%)	44 (28%)	4.1 (2.6–6.5)	6 (14%)	1.7 (0.7–4.1)
**Congestive Heart Failure**	46 (8%)	2 (1%)	0.2 (0.04–0.6)	4 (9%)	1.2 (0.4–3.4)
**Coronary Artery Disease**	73 (12%)	2 (1%)	0.1 (0.02–0.4)	7 (16%)	1.3 (0.6–3.1)
**Hypertension**	141 (29%)	10 (6%)	0.2 (0.1–0.4)	6 (14%)	0.5 (0.2–1.2)
**Congenital Defects**	1 (0%)	1 (1%)	0.4 (0.2–60.7)	0	
**Asthma**	53 (9%)	22 (14%)	1.7 (0.97–2.81)	5 (11%)	1.3 (0.5–3.4)
**COPD**	109 (19%)	17 (11%	0.5 (0.3–0.9)	6 (14%)	0.7 (0.3–1.7)
**Cancer <5 y ago**	81 (14%)	3 (2%)	0.1 (0.04–0.4)	2 (5%)	0.3 (0.07–1.3)
**Cancer >5 y ago**	30 (5%)	3 (2%)	0.4 (0.1–1.2)	0	n/a
**Bone Marrow Transplant**	11 (2%)	0	n/a	0	n/a
**Asplenia**	12 (2%)	0	n/a	0	n/a
**Rheumatoid Arthritis**	15 (3%)	0	n/a	0	n/a
**Systemic Lupus Erythematosis**	13 (2%)	0	n/a	0	n/a
**Chronic Renal Failure**	28 (5%)	2 (1%)	0.3 (0.06–1.1)	2 (5%)	1.0 (0.2–4.1)
**Diabetes**	76 (13%)	5 (3%)	0.2 (0.09–0.6)	6 (14%)	1.1 (0.4–2.6)
**Hypothyroidism**	38 (6%)	2 (1%)	0.2 (0.05–0.8)	1 (2%)	0.3 (0.05–2.5)
**Epilepsy or other seizure disorder**	16 (3%)	3 (2%)	0.7 (0.2–2.4)	4 (9%)	3.6 (1.1–11.2)

14 Missing values assumed non-smoker.

**Table 5:** Description of traits and risk factors among patients 16 years of age or older in a ST5 & ST8 outbreaks, Calgary Health Region 2000–2007 in Multivariate analysis.

	Odds Ratio, Outbreak ST5 cases (2005–2007) N = 156	P Value	Odds Ratio Outbreak ST8 cases (2005–2007) N = 44	P Value
**Demographics**				
**Sex (male)**	1.30	ns^15^	4.39	<0.01
**Age 16–64 years**	2.59	0.06	0.95	ns
**Homeless**	4.41	<0.01	2.58	0.02
**Aboriginal**	0.93	ns	7.34	<0.01
**Pre-existing Risk Factors**				
**Current Smoker** 16	1.32	ns	2.51	0.04
**Alcoholism**	1.50	ns	0.61	ns
**Illegal Drugs**	4.80	<0.01	2.17	0.07
**HIV/AIDS**	0.47	0.11	n/a	
**Hepatitis A or B**	0.87	ns	n/a	
**Hepatitis C**	0.87	ns	n/a	
**Congestive Heart Failure**	0.44	ns	n/a	
**Coronary Artery Disease**	0.36	ns	n/a	
**Hypertension**	0.69	ns	n/a	
**Asthma**	2.63	0.01	n/a	
**COPD**	1.21	ns	n/a	
**Cancer <5 y ago**	0.36	0.11	n/a	
**Chronic Renal Failure**	1.23	ns	n/a	
**Diabetes**	0.40	0.11	n/a	
**Hypothyroidism**	0.85	ns	n/a	
**Epilepsy or other seizure disorder**	n/a	n/a	2.66	ns

15 not significant (P>0.15).

16 Missing values assumed non-smoker.

The ST5 isolates demonstrated phenotypic and genotypic clonality during the outbreak. All isolates were susceptible to penicillin, erythromycin and ceftriaxone and all had intermediate susceptibility to trimethoprim-sulfamethoxazole (TMP-SMX). The MLST analysis of 5 representative ST5 isolates identified that all were sequence type ST289. This is similar to the previously described strain from Columbia also described by the Pneumococcal Molecular Epidemiology Network (Columbia^5^-19 strain) [21], [22], [23].

The ST8 isolates also demonstrated phenotypic and genotypic clonality during the outbreak. All isolates were susceptible to penicillin, erythromycin, ceftriaxone and TMP-SMX. The MLST analysis of 5 representative isolates identified sequence type ST1480 for the first isolate and ST1268 for the remaining 4 isolates.

During the targeted PPV23 vaccine campaign in the homeless shelters , 91 individuals (79 clients and 12 staff) at the main Calgary homeless shelter were enrolled in the nasopharyngeal colonization survey. The questionnaire was completed by 71 clients (89%) and 8 staff (67%). Pneumococcus was isolated from were 15 (16.5%) participants (14 clients, 1 staff). The following serotypes were identified: 28A (isolates); 17F (2 isolates); and 1 each of serotypes 5, 10A 10F, 11F, 12F, 23A, 3 and 38; and 2 isolates were non-typable. Table 6 describes data obtained from the NP colonization survey.

**Table 6:** Clinical and Epidemiologic Features of Clients and Staff at the Drop-In Centre, December 2006, Calgary Health Region.

Epidemiology	Clients (n = 79)	Staff (N = 12) - Questionnaire = 8
**Male Gender**	68 (86%)	7 (58%)
**Average age (years)**	40.7	40.7
**Aboriginal**	34 (43%)	1 (13%)
**Positive nasopharyngeal culture**	14 (18%)	1 (8%)
**Alcohol use**	59 (75%)	
**Tobacco use**	71 (90%)	
**Cocaine use**	10 (13%)	
**Crack cocaine use**	33 (42%)	
**Marijuana use**	28 (35%)	
**Share bottles**	19 (32%)	
**Share cigarettes**	42 (61%)	
**Share crack pipes**	25 (76%)	
**Share joints**	21 (75%)	
**Contact with children** **(total swabs collected = 74 clients** * **)**	16 (20%)	5 (42%)

	Culture positive clients (N = 14)	culture negative clients (N = 60)	P-value (Fisher's exact test (for <5) or Mantel-Haenszel
**Male gender**	13 (92%)	52 (87%)	0.48
**Drop-in centre only (as opposed to other shelters)**	5 (36%)	32 (53%)	0.24
**Aboriginal**	9 (64%)	23 (38%)	0.08
**Alcohol use**	12 (86%)	43 (72%)	0.23
**Tobacco use**	14 (100%)	52 (86%)	0.17
**Crack cocaine use**	6 (43%)	25 (42%)	0.94
**Marijuana use**	7 (50%)	19 (32%)	0.16
**Share bottles**	4 (33%)	13 (30%)	0.41
**Share cigarettes**	8 (57%)	30 (60%)	0.23
**Share crack pipes**	5 (82%)	18 (72%)	0.68
**Share joints**	5 (71%)	14 (74%)	0.34

*7 staff members (1 positive) excluded from the following review.

## Discussion

Outbreaks of *Streptococcus pneumoniae* have been described in closed populations such as hospitals, daycares, schools, military barracks, nursing homes and prisons [1], [2], [3], [4], [5], [6], [7]. The ST5 outbreak in Calgary and across Western Canada is the first one described in a disadvantaged but relatively open community [13]. The outbreak in Calgary area began before the outbreak in Vancouver as described by Romney and colleagues [13]. In addition to the ST5 outbreak, there was an outbreak of ST8 IPD in Calgary that peaked in 2005, the year that an increase in ST5 cases began. This is the first described outbreak of ST8 IPD. Overlapping outbreaks of this kind have not been previously described.

Disadvantaged populations including aboriginals, homeless and impoverished people suffer higher rates of pneumococcal disease [24], [25]. Outbreaks, however, have rarely been described in these populations [13], [26]. Mercat and colleagues described an outbreak in homeless shelters in Paris in the late 1980s due to serotype 1 [26].

Both of the outbreaks occurred predominantly in persons at increased risk for IPD, although the particular factors differed for each serotype. The ST5 cases were more common in homeless persons and those reporting illegal drug use, while the ST8 cases were more common in males, homeless persons and aboriginals. The Calgary homeless population was estimated at 4,060 in 2008 and this number had increased 18% from 2006 [27]. This population is predominantly male (78%), caucasian (62%) with 29% between the age of 45 and 64 [27]. Eighteen percent of individuals in a survey in a subset of the population had been Calgary less than 1 year [28]. Self reported substance abuse in the homeless population was 85% and 24% had mental health issues [28].

Vaccination rates have traditionally been quite low in disadvantaged populations especially those who do not seek regular medical care. In August 2006 the Alberta Advisory Committee on Communicable Diseases recommended offering the 23-valent polysaccharide pneumococcal vaccine to homeless individuals and also those who are hepatitis C positive, and this position was further strengthened by a statement from the National Advisory Committee on Immunization in 2008 [29]. Both of the outbreak strains are be covered by the 23 valent polysaccharide pneumococcal vaccine, however the efficacy of this vaccine is limited [30], [31]. The 7 valent conjugate vaccine (Prevnar®) available at the time of the outbreak does not cover the two outbreak strains [32]. The new 10-valent and 13-valent conjugate pneumococcal vaccines, (the latter now in routine use across Canada), cover these serotypes but are routinely so far only used in pediatric patients [33].

Serotype 5 is a particularly invasive serotype with invasiveness index 60 fold higher than some of the least invasive serotypes (3, 6A, 15) [34]. The ST5 strain has been particularly effective in this outbreak evidenced by the significant presence across Western Canada documented by both our jurisdiction and British Columbia [13]. Serotype 5 was otherwise uncommon in our area among invasive cases and in colonization surveys [35]. Serotype 8 was also uncommon among invasive isolates and accounted for only 1 of 678 colonizing strains identified over multiple years. [35]. However, ST8 is commonly an invasive serotype [34]. The majority of the ST8 sequence types were ST1268, with only the very first isolate tested being slightly different (ST1480 which only differs from ST1268 by 1 allele (*spi*)).

The demographic features, risk factors, clinical course and outcome differed for both ST5 and ST8 cases of IPD, compared with the rest of the adult cases of IPD before, during and after the outbreaks. In addition data from the survey questionnaire during the NP colonization survey several other factors that may be associated with acquisition of pneumococcal disease (Table 6), including cocaine and crack cocaine use, marijuana use as well as the sharing of bottles cigarettes, crack pipes and “joints”. As suggested by Romney, crack pipes and other implements may be a route of transmission for pneumococcus [13].

Although both of the described outbreaks had their onset in the colder months of the year, a clear correlation between seasonality and this outbreak were not well-established (Figures 2 & 3). Crowding in the homeless shelters is more common in the winter months, but shelter use remains significant throughout the year.

Outbreak control measures utilized in the Calgary area included a vaccination campaign in the largest of the homeless shelters, December 2006, that did not result in a rapid resolution of the outbreak. The limited uptake of vaccine, limited vaccine effectiveness, a persistent susceptible population and ongoing crowding may have been factors in the persistence of the outbreak, especially for ST5 disease.

In summary, we describe overlapping outbreaks of ST5 and ST8 in an open homeless community. Significant risk factors that contributed to either or both of these outbreaks included homelessness, illicit drug use, smoking, aboriginal ethnicity and smoking. Targeted vaccination programs, preferably with protein-polysaccharide conjugate vaccines that reduce both pneumococcal disease and transmission, may better control future outbreaks.
